# Associated Mortality Risk of Atypical Antipsychotic Medication in Individuals With Dementia (AMRAAD): A Clinical Cohort Study

**DOI:** 10.1192/bjo.2022.234

**Published:** 2022-06-20

**Authors:** Peter Phiri, Tomas Engelthaler, Hannah Carr, Gayathri Delanerolle, Clive Holmes, Shanaya Rathod

**Affiliations:** 1 Southern Health NHS Foundation Trust, Southampton, United Kingdom; 2 University of Southampton, Southampton, United Kingdom.; 3 Oxford Centre for Innovation, Oxford, United Kingdom; 4 Nuffield Department of Primary Care Health Sciences, Oxford, United Kingdom

## Abstract

**Aims:**

Antipsychotic medications such as risperidone, olanzapine and aripiprazole are used to treat psychological and behavioural symptoms among dementia patients. Current evidence indicate prescription rates for antipsychotics vary and wider consensus to evaluate clinical epidemiological outcomes is limited. This study aims to investigate the potential impact of atypical antipsychotics on the mortality of patients with dementia.

**Methods:**

A retrospective clinical cohort study was developed to review United Kingdom Clinical Record Interactive Search system based data between January 1, 2013 to December 31, 2017. A descriptive statistical method was used to analyse the data. Mini Mental State Examination (MMSE) scores were used to assess the severity and stage of disease progression. A study specific cox proportional hazards model was developed to evaluate the relationship between survival following diagnosis and other variables.

**Results:**

A total sample size of 1692 patients were identified using natural language processing of which, 587 were prescribed olanzapine, quetiapine, or risperidone (common group) whilst 893 (control group) were not prescribed any antipsychotics. Patients prescribed olanzapine and Risperidone showed similar risk of death [hazard ratio (HR) = 1.32; 95% confidence interval (CI): 1.08–1.60; P < 0.01], (HR = 1.35; 95%CI: 1.18–1.54; P < 0.001). Patients prescribed Quetiapine showed no significant association (HR = 1.09; 95%CI: 0.90–1.34; P = 0.38). Factors associated with a lower risk of death were elevated MMSE score at diagnosis (HR = 0.72; 95%CI: 0.62–0.83; P < 0.001) along with other demographic factors such as women (HR = 0.73; 95%CI: 0.64–0.82; P < 0.001) and being of a Caucasian British group (HR = 0.82; 95%CI: 0.72–0.94; P < 0.01).

**Conclusion:**

A significant mortality risk was identified among those prescribed olanzapine and risperidone which contradicts previous findings although the study designs used were different. Comprehensive research should be conducted to better assess clinical epidemiological outcomes associated with diagnosis and therapies to improve clinical management of these patients.

